# From Prediction to Physical Meaning in Materials AI

**DOI:** 10.1021/acsmaterialsau.6c00170

**Published:** 2026-08-31

**Authors:** Meng Li, Pengzhen Lin

**Affiliations:** † Department of Mechanical and Materials Engineering, 4257 Queen’s University, Kingston, Ontario K7L 2N8, Canada; ‡ Department of Statistical Sciences, 7938 University of Toronto, Toronto, Ontario M5S 1A1, Canada

**Keywords:** materials artificial intelligence, physical meaning, claim-evidence relationship, evidence boundary, explainable machine learning, defect chemistry, proton-conducting oxides

## Abstract

Materials artificial intelligence (AI) is increasingly used to support claims that extend beyond property prediction, including physical interpretation, mechanism identification, and design guidance. As the claim shifts from prediction to understanding, the required evidence must shift accordingly. In this Perspective, we argue that physical meaning in materials AI should be treated as a claim-evidence relationship rather than as a property conferred by model architecture or explanation method. Predictive accuracy establishes performance within a tested domain, physical consistency shows compatibility with constraints, and model interpretation reveals how information is used. These are necessary forms of evidence, but they have boundaries, and none by itself justifies a mechanism or design principle. We use a claim-evidence map and validation ladder to identify where evidence boundaries arise and what additional support is required for stronger claims. Examples from out-of-distribution property prediction, text-mined synthesis data, and machine-learned interatomic potentials show how validation domain, data set history, and sampled configuration regime can limit the physical claims a model can support. We then use defect-chemistry-guided materials AI in proton-conducting oxides as a case analysis, where physically defined defect variables allow model outputs to be evaluated across prediction, interpretation, transport mechanism, and domain-limited design descriptors. The central argument is that physical meaning depends on aligning the strength of a materials claim with the evidence available to support it. Progress in materials AI should not be judged by predictive performance alone, but whether models make physical meaning testable.

## Graphical abstract

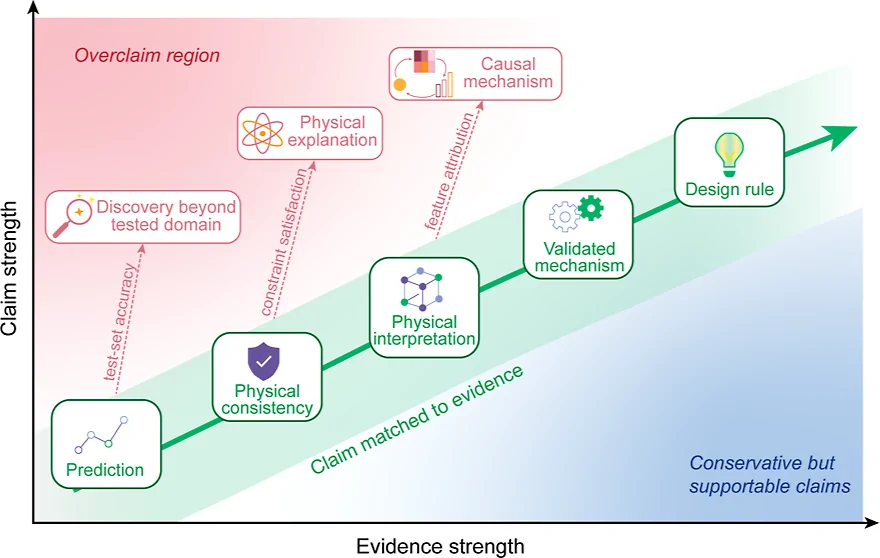

## 1 Introduction

Artificial intelligence (AI) methods now play a substantial role in materials research.^1–3^ Over the past decade, the field has expanded from descriptor-based regression and kernel methods^4,5^ to graph neural networks,^6–8^ equivariant architectures,^9^ machine-learned interatomic potentials,^10,11^ active learning,^12^ autonomous experimentation,^13^ and emerging foundation models.^14,15^ These approaches are now used to predict and screen formation energies,^16^ band gaps, elastic properties, ionic transport, interatomic forces, microstructure–property relationships,^17^ and synthesis routes and conditions.^18,19^ Their impact is not limited to accelerating established workflows. By linking databases, simulations, experiments, and optimization loops, AI methods increasingly shape how materials questions are formulated, prioritized, and tested.

Broad predictive capability, however, does not by itself show that a model has learned transferable physical principles.^20–22^ Materials properties rarely arise from composition or structure alone, but instead emerge from coupled contributions of crystal chemistry, local bonding, point defects, interfaces, microstructure, processing history, and operating environment.^23–26^ A model may accurately predict a test set because it lies near the training distribution, rather than because the model captures a physical principle.^27,28^ This distinction becomes critical when materials AI is used for discovery, mechanism identification, or design-principle extraction, where the model must remain meaningful as chemistry, structure family, defect population, processing route, or thermodynamic condition changes. Recognition of this challenge has motivated two complementary directions. Interpretable and explainable machine learning (ML) methods aim to make model behavior more transparent by identifying descriptors, learned representations, structural motifs, or counterfactual changes associated with predictions.^29,30^ Complementing these efforts, physics-guided, physics-informed, and physics-embedded approaches introduce materials knowledge through physically motivated descriptors, constraint-based loss functions, symmetry- or equivariance-preserving architectures, and workflows coupled to first-principles or physics-based simulations.^9,12,31^ These developments have made models more diagnostic, more data efficient, and less prone to unphysical predictions. They have also moved the field beyond a simple contrast between data-driven fitting and physics-based modeling, setting the stage for a more precise question about when model outputs support claims of materials understanding.^32^

Yet a gap remains. Predictive accuracy, interpretability, and physical consistency can all increase confidence in a materials AI model, but they do not by themselves specify the physical process governing a material response.^27,33^ Constraints such as symmetry, charge balance, stoichiometry, or thermodynamic bounds can penalize or reduce unphysical predictions while leaving the controlling mechanism unresolved. Likewise, feature attribution and learned representations can identify model-sensitive descriptors, structural motifs, or internal features, but they do not establish that those features are causal variables governing the material response.^4^ A highlighted feature may correspond to a real materials variable, such as dopant–defect association, migration barriers, or grain-boundary resistance. It may also act as a surrogate for unmeasured synthesis conditions, processing history, database source, or measurement protocol. This ambiguity is the reason an AI-derived rationale requires independent physical evidence before it can be treated as a mechanism.

This Perspective frames physical meaning as a claim-evidence relationship. Here, physical meaning refers to the extent to which model output is connected to materials variables or observables and supported by evidence appropriate to the claim being made. As shown in Figure 1, a materials AI result should be evaluated according to the strength of the scientific claim attached to it. Predictive performance, physical consistency, and model interpretation can each make a study more credible, but mechanism claims require additional support. A model-derived explanation must be connected to materials descriptors or observables, distinguish plausible physical processes, and be tested through controlled changes in materials variables and independent validation. Recent AI-assisted studies of proton-conducting oxides offer a representative case study for this claim-evidence framework, tracing a progression from hydration-informed prediction and dopant solution energetics to atomistic transport mechanisms, materials design descriptors, and independent experimental validation.^34–36^ Physical meaning is therefore not established by model architecture or explanation method alone. It depends on an evidence chain that connects model output to materials variables, mechanisms, and validation.

**Figure 1:**
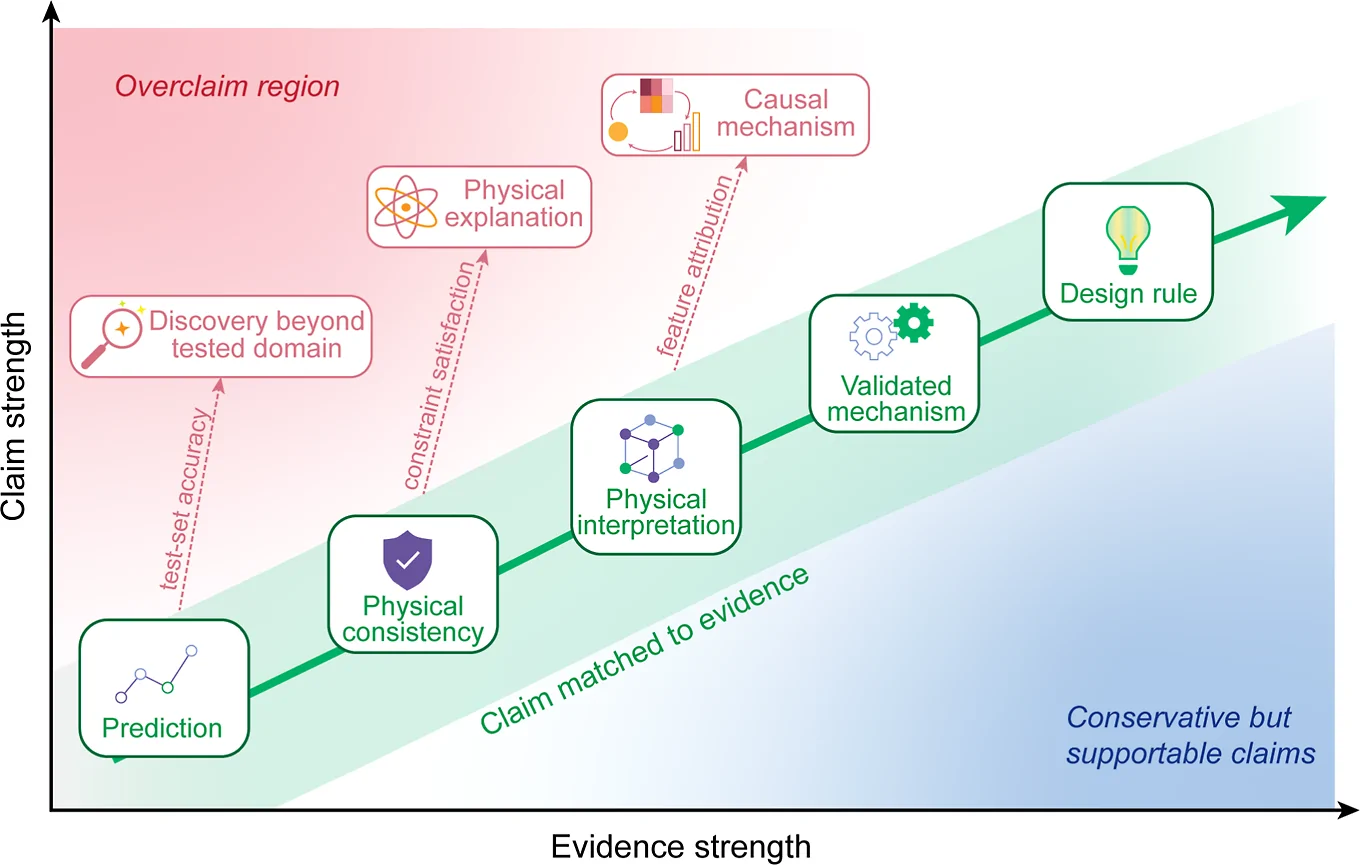
Claim-evidence map for physical meaning in materials AI. Claim strength is plotted against evidence strength. The diagonal path shows claims supported by matching evidence, from prediction and physical consistency to physical interpretation, validated mechanism, and design principle. The upper shaded region indicates overclaiming, and the lower shaded region indicates conservative but supportable claims. Red dashed arrows illustrate representative cases in which a specific form of evidence is used to support a stronger claim.

## 2 Evidence Boundaries in Materials AI

High accuracy in a materials AI model does not automatically justify a mechanistic claim. Benchmark scores, physical constraints, attribution maps, and energy and force errors in atomistic models are all useful, but they test different aspects of a model, including predictive accuracy under a sampling protocol, compatibility with known physical or chemical rules, dependence on learned features or representations, and agreement with reference configurations. These forms of evidence are not interchangeable. A low error on a test set, a charge-neutral output, an attribution that highlights a familiar materials descriptor, or a small energy and force discrepancy can each increase confidence in a model, yet none establishes which physical process dominates a material response. The problem arises when evidence obtained for one purpose is used to support a stronger claim, especially when model performance or physical consistency is interpreted as mechanism. The evidence boundary is reached when the strength of the claim exceeds what that evidence can support, at which point the claim becomes an overclaim.

One clear evidence boundary appears in how test sets are constructed for structure-based materials property prediction. Omee and co-workers compared random cross-validation with sparse structural out-of-distribution (OOD) target selection for graph neural networks, showing that validation design changes what kind of claim model performance can support.^28^ In the random cross-validation distribution shown in Figure 2a, test samples are withheld from training but remain distributed across the same sampled region as the training data. High predictive accuracy under this split supports interpolation within the model’s domain of applicability. In the SparseXcluster construction shown in Figure 2b, target clusters are selected from sparsely sampled structural regions using structure-derived descriptors. Performance under this split provides stronger evidence for transfer to less-sampled regions of materials space. The distinction is that random cross-validation and sparse structural OOD validation support different levels of claim. The former supports prediction within a sampled data distribution, whereas the latter tests whether the model remains reliable when target materials occupy sparsely sampled regions of structural representation space. Neither establishes mechanism unless the prediction is linked to a physical variable whose role can be independently tested.

**Figure 2:**
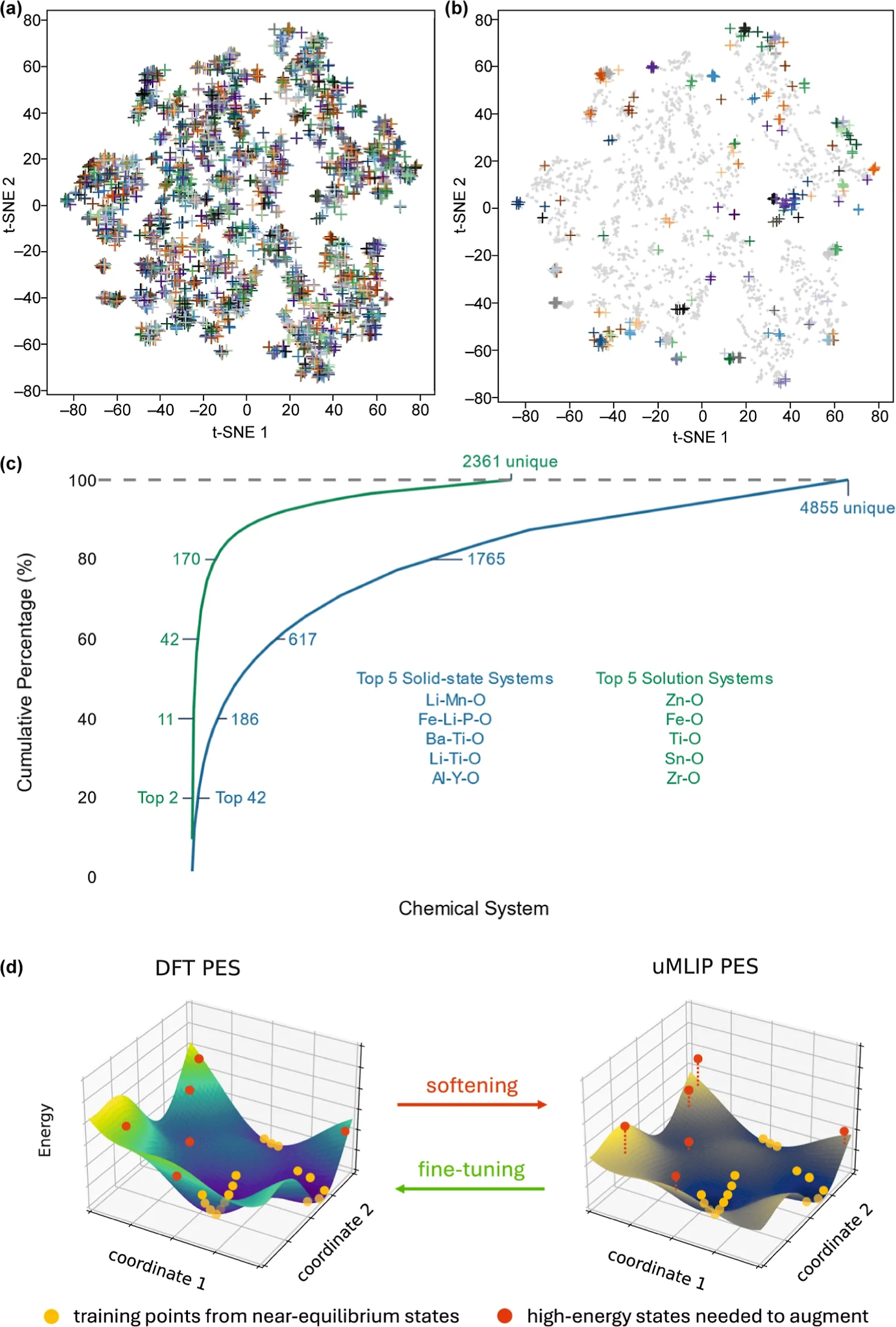
Representative examples of evidence boundaries in materials AI. (a,b) Materials representation-space distributions for random cross-validation and SparseXcluster out-of-distribution target selection. Adapted from ref 28 Available under a CC-BY 4.0 license. Copyright 2024 The Authors. (c) Cumulative distributions of text-mined solid-state and solution-based synthesis recipes over unique chemical systems. Reproduced with permission from ref 39 Copyright [2025] The Royal Society of Chemistry. (d) Potential-energy surfaces (PES) from density functional theory (DFT) and a universal machine-learned interatomic potential (MLIP) for near-equilibrium and high-energy configurations. Reproduced from ref 40 Available under a CC-BY 4.0 license. Copyright 2025 The Authors.

Model interpretation may lead to overclaiming in a less obvious way, because an explanation can be faithful to the data set without identifying the materials mechanism. Feature importance, SHapley Additive exPlanations (SHAP), attention weights, saliency maps, symbolic descriptors, and counterfactuals can reveal how a model uses information.^30,37,38^ They can also identify descriptors, motifs, or processing variables that are physically and chemically plausible. The difficulty is that plausibility is not mechanism. A highlighted feature may correspond to a real materials variable, such as local coordination, dopant–defect association, migration barriers, or grain-boundary resistance. It may also reflect how the data set was assembled, which systems were studied repeatedly, which processing routes became conventional, or which measurements were reported. In that case, the explanation is not necessarily wrong. It is faithful to a pattern in the data, but the pattern may not be the physical origin of the material response.

In synthesis prediction, this ambiguity is especially clear because literature-mined recipes reflect both synthesis chemistry and the uneven record of what has been attempted and reported. Successful routes, familiar precursors, and repeatedly studied chemical systems appear far more often than failed, unpublished, or less-explored alternatives. Sun and David analyzed text-mined solid-state and solution-based synthesis recipes and showed that the reported records are concentrated in a limited number of chemical systems.^39^ The cumulative distributions shown in Figure 2c therefore should not be read only as a data imbalance. They show that synthesis data sets carry a historical sampling structure. A model trained on such records may learn reproducible patterns in precursor choice, temperature range, atmosphere, or processing route. Those patterns may reflect precursor reactivity, intermediate phase formation, thermodynamic accessibility, or kinetic pathway selection. They may also reflect reagent availability, inherited recipes, publication bias, or missing failed syntheses. The evidence boundary appears when such a reported pattern is interpreted as a synthesis mechanism without prospective synthesis, controlled variation of processing parameters, or independent characterization of intermediates and pathways.

Machine-learned interatomic potentials (MLIP) provide a more quantitative version of the same problem. Their training labels, energies and forces, are physically defined, so the temptation to treat model accuracy as mechanistic reliability is especially strong. Deng and co-workers examined universal MLIP and reported systematic softening of the predicted potential energy surface (PES).^40^ In Figure 2d, the universal MLIP reproduces the near-equilibrium region more closely than the high-energy region. This distinction matters because high-energy and sparsely sampled regions of configuration space often contain the events that mechanism claims depend on. Defect migration, ion transport, surface reactions, phase transformations, and mechanical failure involve transition states, distorted local environments, and rare configurations. Agreement near equilibrium can therefore coexist with unreliable predictions in the regimes that control transport, reaction, or failure.

This concern has already reshaped MLIP validation practice. Liu and co-workers showed that conventional energy and force metrics can miss discrepancies in dynamics, defect configurations, and rare events because those environments occupy only a small fraction of typical test sets.^41^ Morrow and co-workers emphasized that validation should be guided by the intended use of the potential.^42^ The relevant test is therefore set by the physical question. For diffusion, migration barriers and transition-state configurations matter more than an average force score. For defect behavior, the validation target shifts to defect formation energies, local relaxations, and near-defect dynamics. Claims about phase stability or degradation place the burden elsewhere, on competing structures, surfaces, phonons, and finite-temperature trajectories. Validation must therefore probe the part of configuration space in which the mechanistic interpretation is made.

## 3 A Validation Ladder for Physical Meaning

Across property prediction, synthesis data, and atomistic modeling, the same pattern emerges. Evidence that is valid for one purpose is often asked to carry a stronger scientific claim. A random split can support prediction within a sampled distribution without testing transfer to sparsely sampled structural space. A model explanation can be faithful to reported data without identifying the materials mechanism behind those data. Close agreement with reference atomistic calculations can validate one configuration distribution without validating the configurations that control transport, reaction, or failure. Table 1 organizes this pattern into a validation ladder for physical meaning. The level of claim is determined not by the algorithm used, but by the relation between the claim attached to a result and the evidence used to support it. The ladder does not rank studies or require every study to progress to its upper levels, but instead aligns claim language with the evidence actually provided.

**Table 1:** Validation Ladder for Physical Meaning in Materials AI^a^

claim level	evidence standard	claim boundary
prediction	appropriate validation, uncertainty quantification, baseline comparison, and domain of applicability	valid within the tested domain
physical consistency	compatibility with symmetry, equivariance, conservation laws, stoichiometry, charge neutrality, thermodynamic bounds, or limiting behavior	physically consistent prediction, not explanation
physical interpretation	model-derived explanation linked to materials descriptors or observables, such as defect energetics, migration barriers, local structure, microstructure, or processing variables	suggests relevant variables, not causality
mechanistic hypothesis	competing mechanisms separated through controlled changes in chemistry, structure, defects, processing, or operating conditions	testable hypothesis, not validated mechanism
validated mechanism	independent experiment, simulation, or characterization supports the proposed mechanism	case-specific mechanism unless transfer is tested
design principle	a validated mechanism guides new materials across chemistry, structure, processing, or operating regimes with stated boundary conditions	domain-limited principle

a Evidence standards for increasingly strong claims, from prediction to design principle.

At the lower end of the ladder, validation defines the domain in which a result is supported. Robustness tests on newly added Materials Project entries by Li and co-workers,^43^ the structure-based OOD benchmark of Omee and co-workers,^28^ and the representation-space analysis of OOD generalization by Li and co-workers^27^ all make the validation domain visible in different ways. In small-data degradation modeling, Cheng et al. bring out the same issue in an application-driven setting, where sparse and heterogeneous measurements make the domain of applicability especially narrow.^44^ These studies support prediction as a meaningful scientific result, but they also limit what prediction can claim. Physical consistency strengthens this result when governing equations, constraints, physical priors, graph representations, or equivariant architectures are built into a model, as illustrated by the physics-informed learning framework of Karniadakis et al.,^45^ the materials graph-neural-network review of Reiser and co-workers,^46^ and Ghosh’s discussion of physics-informed, explainable, and causal models for materials research.^47^ The added physical structure can improve plausibility and data efficiency, but it does not identify the material process responsible for the response.

Interpretation raises a different claim because a model-derived signal can be physically plausible without being mechanistic. Reviews of interpretable and explainable machine learning in materials science by Oviedo et al. and Zhong et al. place feature attribution, learned representations, attention-based analysis, and descriptor-based explanations in the context of model diagnosis and hypothesis generation.^29,30^ Zhou and co-workers’ survey of explanation quality adds a useful distinction between explanations that are plausible, faithful, robust, or useful.^48^ Counterfactual molecular explanations, as developed by Wellawatte and co-workers, illustrate how model explanations can be turned into testable alternatives when the proposed changes are chemically meaningful.^49^ These works establish the value of interpretation, but they also define its boundary. A descriptor, motif, synthesis variable, or learned feature becomes physically interpretable only when it can be tied to a materials quantity that can be computed, measured, or modified. It becomes mechanistic only when changes in that quantity distinguish one physical explanation from plausible alternatives. This distinction is essential to the broader argument by Krenn et al. that AI-enabled prediction does not automatically constitute scientific understanding.^20^

Claims beyond interpretation require evidence outside the original model, and design claims require transfer beyond the initial case. MLIPs provide a useful methodological precedent because their validation has already confronted the gap between average accuracy and mechanism-level reliability. Investigations on MLIP validation have shown why average energy-force agreement is insufficient for mechanism-level claims in rare-event, defect, or high-energy regimes.^40–42^ The lesson is that validation must reach the physical regime in which the mechanism is asserted. A validated mechanism may remain specific to the material system and conditions in which it was established, whereas a design principle must guide new materials within stated chemical, structural, processing, or operating boundaries.^50–52^ Examples include hydration-informed perovskite search by Hyodo et al.,^34^ defect-chemistry-trained host-dopant selection by Fujii et al.,^35^ analysis of proton transport and lattice softness in heavily Sc-doped perovskites by Tsujikawa et al.,^36^ and Li and co-workers’ oxygen-vacancy prediction for perovskite electrocatalysts.^53^ Sun and David’s analysis of text-mined synthesis recipes provides the complementary caution. Reported recipe patterns can be physically meaningful, but they can also reflect precursor availability, community practice, reporting bias, or inherited processing routes.^39^ Accordingly, transfer from a validated mechanism to a design principle must be supported within an explicit domain rather than inferred from a successful model or observed pattern alone.

## 4 Tracing Physical Meaning in Defect-Chemistry-Guided Materials AI

Defect-chemistry-guided materials AI provides a useful case for applying the claim-evidence ladder because model outputs can be compared with measurable defect populations, computed energetics, transport barriers, and operating-condition-dependent measurements. Proton-conducting oxides are especially suitable for this analysis because their transport behavior is shaped by coupled processes including acceptor incorporation, hydration-driven proton uptake, dopant–proton association, proton migration, grain-boundary resistance, and phase stability under operating atmospheres.^54^ A trend associated with dopant identity or dopant concentration may therefore reflect carrier generation, proton trapping, mobility changes, microstructural resistance, or stability limits, each of which implies a different design action. The question is not whether AI can rank compositions, but whether its predictions and interpretations can be connected to defect-chemical quantities that can be computed, measured, or deliberately changed. This connection separates composition-level correlation from physical interpretation, mechanism, and bounded design principle.

The first claim-level shift occurs when prediction is tied to a defect-chemical requirement rather than to composition alone. In the work of Hyodo et al., the descriptor-space maps in Figure 3a do not simply rank oxide compositions by an empirical performance label.^34^ They organize candidate perovskites around hydration-related thermodynamic quantities, making proton incorporation part of the prediction problem itself. Fujii and co-workers extend this logic by combining *ab initio* hydration and dopant solution energetics with interpretable machine learning, materials selection, synthesis, and conductivity measurements, as summarized in Figure 3b.^35^ The important advance is that the model interpretation is constrained by defect-chemical requirements rather than by composition alone. A viable proton-conducting oxide candidate should satisfy both defect-chemical criteria, such as favorable hydration thermodynamics and feasible dopant incorporation, and practical criteria for synthesis and measurement. This evidence moves the claim from composition-level prediction to defect-chemical interpretation and candidate selection. It does not yet establish a transport mechanism, because hydration thermodynamics and dopant solution energetics address whether protons can be incorporated and dopants can be accommodated, but do not by themselves establish proton mobility or the trapping and migration pathways that govern transport.

**Figure 3:**
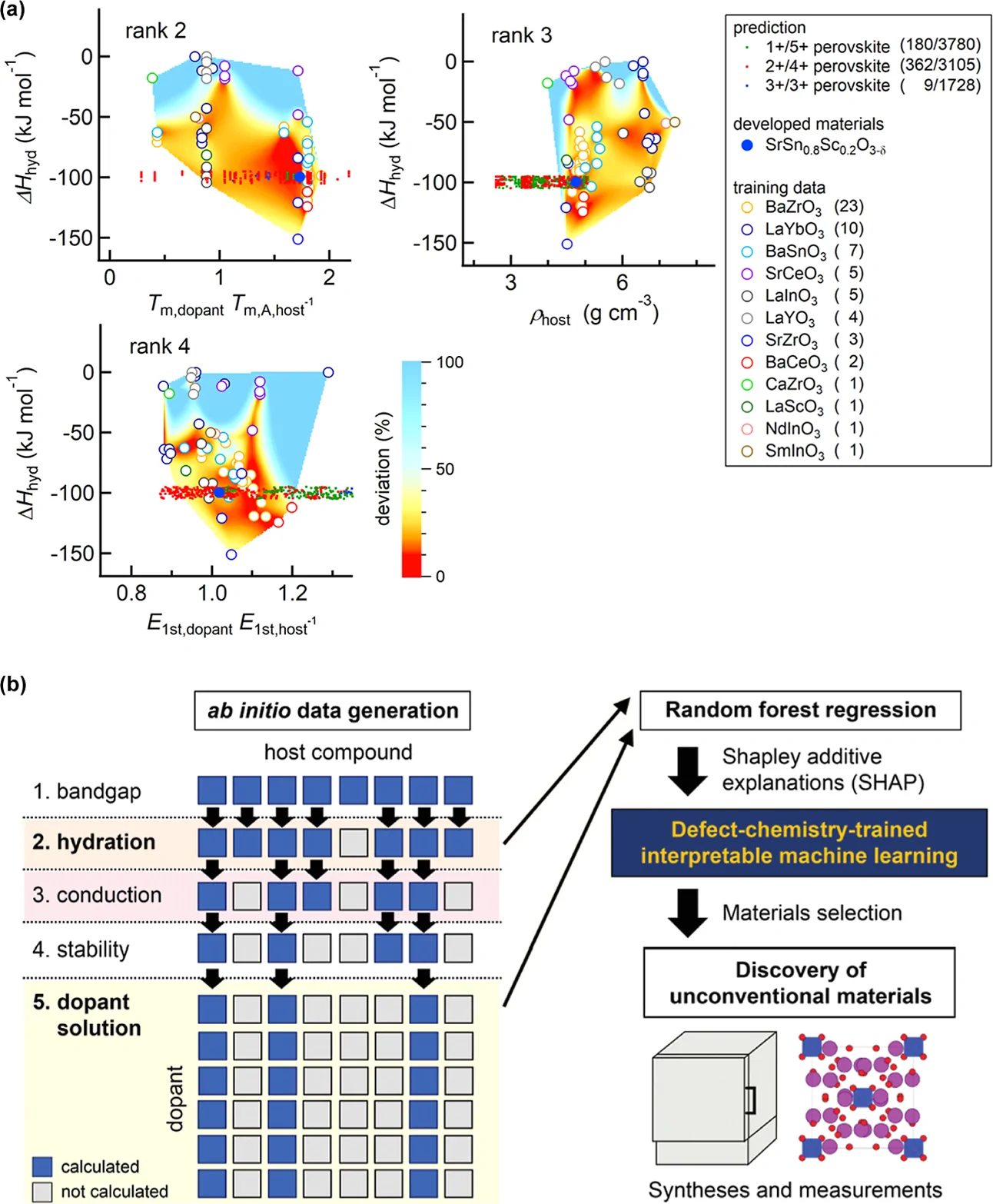
Defect-chemistry-guided prediction and interpretable learning for proton-conducting oxides. (a) Hydration descriptor-space maps used to identify proton-conducting perovskite candidates from hypothetical compositions. The colored scale indicates prediction deviation in descriptor space, open circles denote training compounds, and colored markers denote predicted candidate compositions. Reproduced from ref 34 Copyright [2021] American Chemical Society. (b) Defect-chemistry-trained interpretable machine-learning workflow linking *ab initio* data generation with random forest regression, Shapley additive explanations, materials selection, synthesis, and measurements. Reproduced from ref 35 Available under a CC-BY 4.0 license. Copyright 2023 The Authors.

The mechanism-level question emerges when defect-chemical plausibility must be separated from transport control. In heavily acceptor-doped proton conductors, increasing dopant content can increase the concentration of charge-compensating defects, but it can also strengthen dopant–proton association and reduce mobility.^55^ Tsujikawa et al. address this tension in heavily Sc-doped perovskites by combining machine-learning force-field molecular dynamics with local migration analysis and transport measurements.^36^ The proton-density distribution in Figure 4a provides a transport picture in which proton motion is organized along connected ScO_6_ octahedral environments. This interpretation is stronger than a visual correlation because it is tested against dopant-specific migration behavior, separating the proposed Sc-mediated transport pathway from a simpler carrier-concentration argument. In the claim-evidence ladder, this is the step at which a physically interpretable variable begins to support a mechanistic hypothesis.

**Figure 4:**
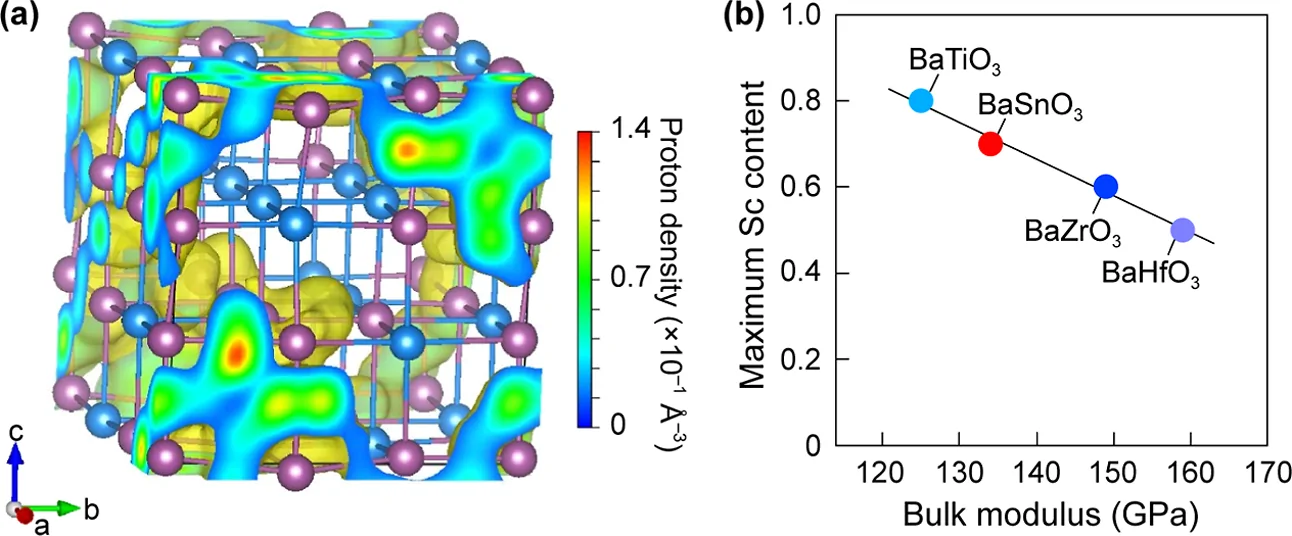
Proton-transport pathway and lattice-softness descriptor in heavily Sc-doped proton-conducting perovskites. (a) Proton-density distribution from machine-learning force-field molecular dynamics, showing proton transport along connected ScO_6_ octahedral networks. (b) Maximum Sc content as a function of bulk modulus in Ba-based perovskite oxides, linking lattice softness to high Sc incorporation. Adapted from ref 36 Available under a CC-BY 4.0 license. Copyright 2025 The Authors.

The design-level question is whether a proposed mechanism can guide host-lattice selection within a defined materials family. Figure 4b connects the heavily Sc-doped transport picture to a design descriptor by relating maximum Sc content to bulk modulus in Ba-based perovskite oxides. Lattice softness is not proposed as a universal rule for all proton conductors. Its value comes from a specific mechanistic context in which a softer lattice can accommodate high Sc incorporation and enable the connected local environments implicated in proton transport. This is the difference between a correlation that describes known compounds and a bounded design principle. The relation between bulk modulus and maximum Sc content gains design value only when interpreted through the Sc-incorporation and transport mechanism within Ba-based perovskites. Outside this host-lattice materials family and the high-Sc-incorporation regime, lattice softness remains a hypothesis to be tested rather than a transferable rule.

## 5 Summary and Outlook

Materials AI increasingly supports claims that go beyond property prediction, including interpretation, mechanism identification, and design guidance. The key question is therefore not which model class is more advanced, but what evidence is needed for a given claim to carry physical meaning. Predictive accuracy, physical constraints, and model interpretation can strengthen a study, but they do not establish mechanism unless the model output is linked to a measurable or computable materials variable, distinguishes plausible alternatives, and is validated in the relevant physical regime. This claim-evidence relationship is the core argument of this Perspective. It preserves the value of prediction, physical consistency, and interpretation while preventing them from being overstated as mechanism or design principle.

The practical route forward is to design materials AI workflows around mechanism discrimination. Instead of optimizing only for lower prediction error or more visually plausible explanations, models can be asked to identify the variables that would most clearly separate competing physical mechanisms. In active learning, this means selecting compositions, defect concentrations, strain states, atmospheres, temperatures, microstructures, or atomic configurations because they are maximally informative about mechanism, not only because they reduce property uncertainty. In atomistic modeling, training and validation should emphasize the configurations where the claimed mechanism occurs, including defects, interfaces, transition states, rare events, and finite-temperature trajectories. In synthesis and microstructure studies, model explanations become more useful when paired with processing metadata, failed trials, controlled variations, and independent characterization, so that correlations in the data set can be separated from variables that dominate the material response. These changes do not require a new category of materials AI. They require a shift in objective, from predicting the next candidate to identifying the next physical test.

How progress in materials AI is evaluated should shift from predictive performance alone to whether models make physical meaning testable. A materials AI result should not be judged only by predictive accuracy, search scale, or the apparent plausibility of its explanation, but by whether it reduces the physical ambiguity that remains after prediction. A result becomes materials knowledge when it identifies the physical process proposed to govern the response, the evidence that can separate it from alternatives, and the limits of its validity. This level of evidence is higher than accuracy, interpretability, or physical consistency alone, but it is required when AI is used to claim explanation, discovery, or design.
